# Systematic review and meta-analysis of quotation inaccuracy in medicine

**DOI:** 10.1186/s41073-025-00173-z

**Published:** 2025-07-23

**Authors:** Christopher Baethge, Hannah Jergas

**Affiliations:** 1 Department of Psychiatry and Psychotherapy, Faculty of Medicine, University of Cologne, Kerpener Str. 62, Cologne, 50937 Germany; 2 Deutsches Ärzteblatt & Deutsches Arzteblatt International, Editorial Offices, Cologne, Germany; 3 Department of Neurology, Faculty of Medicine, University of Cologne, Cologne, Germany

**Keywords:** Quotation inaccuracy, Citation errors, Secondary quotations, Quotation errors, Medical publishing

## Abstract

**Background:**

Quotations are crucial to science but have been shown to be often inaccurate. Quotation errors, that is, a reference not supporting the authors’ claim, may still be a significant issue in scientific medical writing. This study aimed to examine the quotation error rate and trends over time in the medical literature.

**Methods:**

A systematic search of PubMed, Web of Science, and reference lists for quotation error studies in medicine and without date or language restrictions identified 46 studies analyzing 32,000 quotations/references. Literature search, data extraction, and risk of bias assessments were performed independently by two raters. Random-effects meta-analyses and meta-regression were used to analyze error rates and trends (protocol pre-registered on OSF).

**Results:**

16.9% (95% CI: 14.1%-20.0%) of quotations were incorrect, with approximately half classified as major errors (8.0% [95% CI: 6.4%-10.0%]). Heterogeneity was high, and Egger’s test for small study effects remained negative throughout. Meta-regression showed no significant improvement in quotation accuracy over recent years (slope: -0.002 [95% CI: -0.03 to 0.02], *p* = 0.85). Neither risk of bias, nor the number of references were statistically significantly associated with total error rate, but journal impact factor was: Spearman’s ρ = –0.253 (*p* = 0.043, binomial test, *N* = 25).

**Conclusions:**

Quotation errors remain a problem in the medical literature, with no improvement over time. Addressing this issue requires concerted efforts to improve scholarly practices and editorial processes.

**Supplementary Information:**

The online version contains supplementary material available at 10.1186/s41073-025-00173-z.

## Introduction

Citations are a defining element of scientific communication. They support the authors’ assertions, refer readers to earlier work, and give credit to other scholars. As a collaborative effort, science is based on trust [1] in what fellow scientists have contributed and written, including trust in their references to the literature. Citing is so central to the conduct of science that the most important quantitative measures of scholarly achievement, e.g., the impact factor or the h-index, are based on citations.

“Citation” often is used generically for all references to a source, but in this context, a distinction has been established between citation and quotation inaccuracy: Citation inaccuracy usually denotes technical errors, e.g., misspelled author names or wrong page references. In contrast, quotation inaccuracy signifies errors of content: a claim made by the authors quoting the source misrepresents the source. The present investigation is solely concerned with quotations.

Ideally, quotations are correct. And yet, in an early investigation into their accuracy, De Lacey et al. reported substantial error rates in major medical journals [2]. Ever since, dozens of studies have confirmed this finding (e.g., [3–6]). Summarizing research [7–9] arrived at quotation error rates of one in four to seven, with half of all misquotations considered major, that is, the claim is not at all supported by the source.

The most recent review [9], however, is based on only fifteen studies, and the most comprehensive systematic review and meta-analysis [8] included studies published until 2014. In the meantime, new studies have been published e.g., [3, 4, 10]. Also, it is hoped that error rates have improved after misquotations received broad attention in scholarly publishing [11–13]. Therefore, we updated our earlier meta-analysis [8] to estimate current error rates and to look for an improvement in recent years.

## Methods

This systematic review and meta-analysis has been conducted and is presented according to the Cochrane Collaboration Handbook [14] and the PRISMA statement (Preferred Reporting Items for Systematic Reviews and Meta-Analyses [15], eTable 1). It has been pre-registered in the Open Science Framework (OSF).

We followed the literature in differentiating total, major, and minor quotation errors: While there is no universally agreed upon definition, major errors usually mean that a quotation seriously misrepresents the source; it is not at all in accordance with the authors’ claim [2, 8]. For example, De Lacey et al. classified as major errors statements that made strong claims—such as that “immediate memory span is intact” in Korsakoff’s syndrome—based on sources that did not mention the condition at all. Minor errors are usually defined as inconsistencies and factual errors not severe enough to contradict the claim by the authors [16], e.g., a small error in an incidence rate. An example given by De Lacey et al. is a quotation that referred to “42 patients” when the original source had actually described “42 abscesses in 40 patients.” Secondary quotations are quotations of a source that contained a quotation to the source of interest, for example a narrative review article quoting an original paper on the incidence of a condition. Many authors also consider secondary quotations as errors because 1) readers do not find the claim in its original form in the referenced source; 2) authors of review articles get the credit but not the researchers producing the data; 3) secondary quotations have high total error rates themselves [17]. In defining total, major, and minor quotation errors and secondary quotations, we followed the authors of the studies selected.

### Eligibility criteria

We searched for all studies investigating quotation error rates in the medical literature, including but not restricted to quotation errors in journal articles. No date or language restrictions applied. As recommended by PRISMA 2020 [15], the PRISMA-S extension [18], and the AMSTAR-2 [19] tool for systematic reviews and meta-analyses, we included grey literature (e.g., dissertations, conference abstracts) to minimize publication bias and enhance transparency. Owing to the danger of selection bias we excluded studies limited to errors in quotations to single papers. Finally, we also excluded studies on quotation accuracy in advertisements or on the web.

### Literature search

We updated our meta-analysis from 2015 [8] that included studies published until December 26, 2014, by literature searches in Medline and PubMed Central via Pubmed and in Web of Science from January 1, 2014, until October 1, 2023. We hand-searched all reference lists of included papers (search details in eTable 2). After screening titles and abstracts, all potentially eligible studies were read as full-texts. Screening and full-text searches were carried out by at least two independent reviewers (HJ, AH), and studies were selected after discussion among all researchers (HJ, AH, CB).

### Data collection

Two reviewers independently documented data from the papers in a pre-specified Excel spreadsheet, including study meta-data, methods applied, observation period (i.e., the date when quotations to the sources were made), medical field, risk of bias (see below), and all results regarding total, major, minor, and secondary errors.

We collected data on the number of quotations or references investigated and on the number of errors found among those quotations or references. We preferred results on all quotations in the texts in the primary studies because, often, authors quote a reference more than once [8]: In many studies, the number of quotations analyzed exceeds the number of references under scrutiny. Several studies, however, provide only the number of references checked, not the number of quotations. For such studies, calculating quotation errors on the basis of the number of references may inflate error rates. We therefore imputed the number of quotations based on the average number of quotations to a reference as it was reported in all studies providing this information. This approach ensures a more accurate denominator, as it accounts for the fact that references are often cited multiple times within a paper.

In case of unclear or missing data, we approached the corresponding author by e-mail.

### Risk of bias

Absent a risk of bias tool specifically designed for original research on quotation inaccuracy, we revised the Joanna Briggs Institute (JBI) Critical Appraisal Checklist for Studies Reporting Prevalence Data [20] for the purposes of this study (eTable 3).

In bivariable meta-regression we searched for an association of quotation error rates and score on the revised JBI checklist, and we dichotomized studies at the median of 7 into low (> 7) versus high (≤ 7) risk of bias (RoB) studies and compared differences between groups.

### Effect measures

The co-primary outcomes are 1) the summary estimate of total quotation error rates 2) the time trend of total quotation errors analyzed in a maximum likelihood meta-regression across the observation period covered in the studies included. Total errors include major and minor errors and secondary quotations. Secondary outcomes are major and minor errors and their time trends.

### Data synthesis

Summary quotation error rates and 95% confidence intervals were calculated in random-effects meta-analysis based on logit transformed data and DerSimonian & Laird tau^2^ estimators [21]. Both are standard approaches and allow comparisons with earlier work (e.g. [8]). In light of the discussion about preferrable data transformations and tau^2^ estimators for proportions, we re-calculated all random-effects summary estimates based on the arcsine square root data transformation and on the Sidik & Jonkman tau^2^ estimator [22] (sensitivity analysis).

Heterogeneity measures are provided as I^2^ statistic, tau^2^, and 95% prediction intervals.

In addition to study quality (RoB) and observation period we investigated in a subgroup analysis whether medical specialty, defined as surgical versus non-surgical versus mixed/not clinical, was associated with quotation errors. Another potentially predictive factor defined apriori was impact factor (IF): We calculated correlation coefficients (Spearman’s ρ) from journal-specific total error rates and IFs, computed a weighted summary correlation coefficient (including Fisher-z-transformation and back-transformation), and tested for statistical significance in a binomial test.

### Publication bias

Small study effects, as a signal of publication bias, were analyzed by visual inspection of funnel plots, in Egger’s regression test, and in a trim-and-fill procedure (Duval & Tweedie).

All calculations were carried out in Excel, Comprehensive Meta-Analysis 2 and 4, and in metaHUN.

### Deviations from the protocol

We had planned to provide the percentage of articles with at least one quotation error, but many studies did not include this figure so that there is little added value for interpretation. We thus did not conduct this analysis. Errors in quoting a single paper and quotation errors from fields outside medicine are not the focus of this analysis.

## Results

Out of 5094 database records screened and 52 study reports assessed as full texts we included 18 reports on 18 new studies [3, 4, 10, 23–38], for a total of 46 studies – together with 28 studies [2, 3, 5, 6, 16, 17, 17, 39–45, 45–60] from our systematic review published in 2015 [8] – that form the basis of this meta-analysis (see PRISMA flowchart in eFigure 1). We neither selected studies from other sciences than medicine nor papers on quotations of single sources [61, 62].

Studies were published between 1985 and 2024, covered quotations published 1984-2022, and came from 14 countries, predominantly from the US (19) and the UK (9). They originated with 22 medical fields, including general medicine (8 studies), surgery (7), and psychiatry (4). Most investigations focused on quotations in English, but some included articles and quotations in Dutch, German, and Chinese. Across all studies, a total of 32,074 quotations were investigated, derived from references across a median of 235 citations per study (range 43–5973). The average number of quotations per reference was 1.56 (standard deviation [SD] 0.25), calculated from 14 studies in 13 papers providing numbers [5, 6, 10, 17, 29–31, 36, 42, 43, 47, 52, 63]. For 22 studies we imputed the denominator as described in Methods. Table e4 gives study characteristics, including risk of bias (RoB). Seven studies presented agreement figures of error assessment by raters for an average kappa of 0.73 (range: 0.61–0.96) [33, 42, 43, 46, 52, 56, 63], indicating good interrater-agreement.

### Percentages and time trends of total, major, and minor errors

**Fig. 1:**
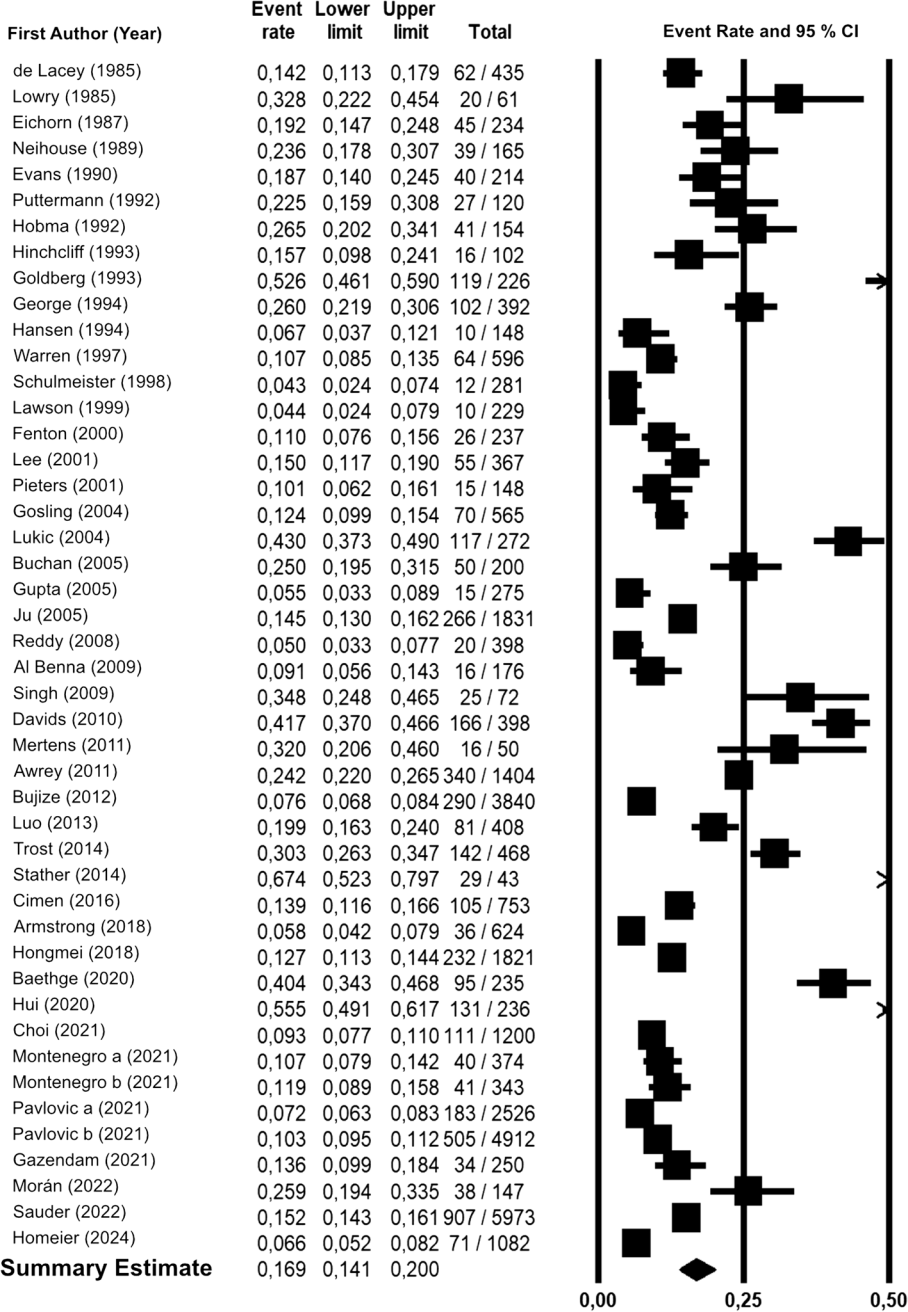
Forest plot of total quotation errors. Legend: Forest plot of total quotation error numbers in all studies included. Horizontal lines indicate 95% confidence intervals, and black boxes denote the mean. The black diamond at the bottom shows the summary effect with 95% confidence intervals

46 studies provided data on total quotation errors, with a summary estimate of 16.9% [95% confidence interval (CI) 14.1–20.0] (Fig. 1). Major errors have been reported in 36 studies and the summary estimate amounted to 8.0% [6.4–10.0] (eFigure 2), while the estimate for minor errors (36 studies), was 7.8% [5.7–10.5] (eFigure 3). The percentage of secondary quotations was 5.3% [3.3–8.5] (18 studies) (eFigure 4). Heterogeneity was high throughout analyses (Table 1 gives main results and heterogeneity figures).

**Table 1:** Main results and heterogeneity estimates

	Summary estimate, %	95%-confidence interval, %	95%-prediction interval, %	I^2^, %	tau^2^
Quotation error (studies)					
Total errors (46)	16.9	14.1–20.0	5–46	98	0.493
Major errors (36)	8.0	6.4–10.0	2–26	94	0.461
Minor errors (36)	7.8	5.7–10.5	1–38	97	0.911
Secondary errors (18)	5.3	3.3–8.5	1.0–36.0	97	1.109

**Fig. 2:**
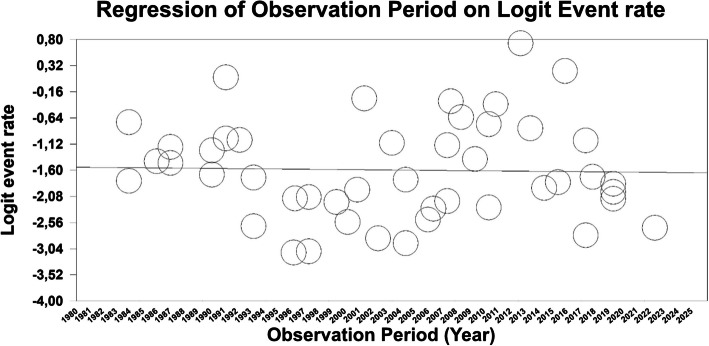
Bubble plot: Meta-regression of time of observation on total quotation errors. Legend: Regression of observation period (in years) against the logit-transformed event rate based on the total quotation error rates reported in each study. Each circle represents a study. The y-axis denotes the logit-transformed event rate, while the x-axis represents the year of the observation period (or the mean in case of more than one year). The horizontal trend line indicates no significant association between the observation period and the logit event rate, suggesting no improvement or decline in event rates over time

Meta-regressions showed constant quotation error rates across the period covered. This applied to all categories: total errors, with a slope of −0.002 [−0.03–0.02], *p* = 0.85 (Fig. 2), major errors: 0.002 [−0.02–0.03], *p* = 0.89 (eFigure 5), minor errors: −0.01 [−0.04–0.02], *p* = 0.44 (eFigure 6), and secondary quotations: −0.01 [−0.05–0.03], *p* = 0.55 (Figure e7).

### RoB

Median risk of bias score was 7 out of 9 points (range 3–9) on the adapted JBI checklist (*N* = 46 studies). 19 had low RoB (> 7 points). In univariable meta-regressions, RoB score was not associated with total (slope: −0.11 [−0.27–0.04], *p* = 0.14), major (slope: −0.03 [−0.2–0.2], *p* = 0.79), minor (slope: −0.1 [−0.3–0.1], *p* = 0.33), or secondary quotation errors (slope: −0.2 [−0.5–0.2], *p* = 0.28), neither was RoB defined categorically (above versus equal or lower to 7; data not shown).

### Publication bias

Egger’s test remained negative across all levels of errors, with no p-value below 0.10, not indicative of publication bias. Based on the inspection of funnel plots, no figures representing *lower* error rates needed to be considered in trim and-fill-procedures, but *higher* error rates were added to the funnel plots of total errors (4 potential studies for a summary estimate of 18.7% [15.7–22.0]), major errors (*n* = 7, 9.8% [7.9–12.1]), minor errors (*n* = 3, 8.8% [6.6–11.8]), and secondary errors (n = 1, 5.9% [3.6–9.3]) (eFigure 8).

### Subgroup analyses

There were no statistically significant differences among surgical, non-surgical, and mixed medical specialties in total errors. (*p* = 0.90, Q-test, df: 2). Similar results emerged for major and secondary quotations errors, while minor errors differed: surgical: 5.0% (14 studies), mixed: 8.8% (*n* = 13), and non-surgical: 13.0% (*n* = 9), p = 0.007 (Q-test, df: 2).

In a pre-planned analysis, total quotation error rate was found to be statistically significantly (p = 0.043, binomial test, N = 25) negatively correlated with journal impact factor: Spearman’s ρ = –0.253 (based on 23 studies reporting correlation coefficients).

*Posthoc*, we found 14 studies without association of quotation errors with the number of references with error rates versus two with an association.

Comparing studies with (*n* = 22) versus without (*n* = 24) imputed denominator revealed statistically significantly lower total quotation error rates in the former: 12.7% [9.8–16.4] versus 21.5% [16.8–27.0], *p* = 0.003 (Q-test, df: 1). The same applied to major errors: 6.1% [4.4–8.4] in 19 imputed versus 10.5 [7.7–14.2] (*p* = 0.017) in 17 unimputed studies, and minor errors: 5.4% [3.5–8.3] versus 11.2% [7.3–16.9] (*p* = 0.019) but not in secondary quotations: 5.4% [2.3–12.4] and 5.2% [2.8–9.5] (*p* = 0.94). When we re-calculated a summary estimate restricted to the 28 studies used in our earlier meta-analysis [8] but with all imputations employed in the current analysis we arrived at a total error rate of 17.0% [13.0–21.9].

### Sensitivity analysis

Re-calculating all analyses based on arcsine square root transformations or/and with Sidik & Jonkman’s tau^2^ estimator generated values from 0.1% below to 1.3% above estimates of quotation errors in initial analyses (eTable 5).

## Discussion

This study yielded three main results: Firstly, the meta-analysis yielded a total quotation error rate of 16.9%. Among these, 8.0% were classified as major errors and 7.8% as minor errors, while the rate of secondary errors was 5.3%. These findings indicate that approximately every sixth quotation is incorrect to some degree, and every twelfth to thirteenth quotation is entirely unsupported by the cited source. Secondly, error rates remained constant since quotation error research began in the 1980s, particularly, it has not improved in the last ten years. Thirdly, rates vary but they are consistently found in a wide variety of medical texts and specialties, as well as in journals of diverse scientific impact and languages.

### Validity of results and study limitations

Have researchers been too rigid in determining authors’ errors, have the methods applied produced invalid results? There is no gold standard in assessing quotation errors, and personal judgement is always involved. However, in our experience, double-checking quotations is often straightforward. This can be seen in many examples presented in quotation inaccuracy studies. Also, the majority of studies employed a) clear and practicable definitions of what constitutes an error, and b) two independent raters. And, at least in the studies providing figures, kappa averaged at 0.73, indicating good interrater agreement according to Landis and Koch, [64] although agreement may be better for major than for minor errors [56].

The findings have proven robust in sensitivity and subgroup analyses: 1. Egger’s tests were not suggestive of publication bias, but when we tentatively tried to factor in possible unpublished studies in trim-and-fill-analyses, figures increased rather than decreased. 2. Other ways of calculating random-effects meta-analyses arrived at similar or higher estimates. 3. There is no indication that high error rates result from low-quality science: two sufficiently powered risk-of-bias analyses remained negative. Also, in contrast to our earlier meta-analysis [8] and similar to Mogull’s approach [9], we erred on the conservative side in calculating a denominator that included the virtual number of possible quotations. When we re-calculated the meta-analysis from 2015 with imputed data, we arrived at a figure very similar to our current overall result (total quotation error rate of 17.0% [12.9–22.0], *n* = 28). In 22 studies with imputed quotation denominator, error rates were lower than in studies providing enough data, suggesting this study's approach is conservative. It is also possible that one quotation is wrong in more than one respect. Five studies provided numbers, for a mean factor of 1.15 errors per index quotation error [17, 30, 58, 60], but many reported merely the first error of a quotation. Also, no study presented results on an error type harder to investigate: The lack of a quotation where one should have been given. Taken together, our figures are more likely an under- than an over-estimation.

It is possible we have missed studies. For example, we could not retrieve some Chinese papers. However, there was no language restriction in our search, we included studies on quotations in other Chinese as well as in Dutch and German articles, and with 46 studies the findings rest on a solid base.

The more than 30,000 references and quotations analyzed across the included studies stem from a wide variety of journals, cover different publication periods, and span nearly all areas of medical research. While we cannot entirely exclude the possibility that a specific quotation was assessed in more than one study, such overlap presumably has occurred only rarely and does not represent a meaningful threat to the validity of our meta-analytic results.

High heterogeneity and wide prediction intervals reflect that single studies may report vastly different error rates. For statistical reasons, high heterogeneity is more likely to appear in single group meta-analyses than in meta-analyses of group comparisons [65] and thus our finding is not surprising nor in contrast to our first meta-analysis [8]. It is prudent anyway, not to rely on the exact point estimates reported here. The CIs, on the other hand, are reasonably small to allow for an estimate of the range of plausible results. Besides, most studies, while technically heterogeneous, are similar in reporting relatively high error rates: More than three thirds found total quotation error rates of more than 10% – they are homogenous in calling our attention to too many errors.

If we tentatively apply GRADE criteria to our primary results, we do not see an effect of RoB or publication bias, neither is there indirectness because studies stem from a wide range of fields and represent different forms of medical texts. However, there is heterogeneity (inconsistency) in our data, but not to the point where a sizable subgroup of studies indicated quotation errors were not a problem and could be called negative. Finally, while point estimates have to be viewed as preliminary (imprecision), confidence intervals show sufficient precision. We thus grade the certainty of the effects to be moderate to high.

Most included studies focused on quotations within original peer-reviewed research articles. Reviews, editorials, and grey literature were rarely examined and were therefore not systematically analyzed. This may be relevant, as quotation practices and the consequences of inaccuracies could differ depending on the publication type. Also, some quotation errors may result from manuscript revisions where references are not updated accordingly, a process-related issue rather than scholarly misconduct. Furthermore, this study is not concerned with the appropriateness or selectivity of citations. Authors may preferentially cite sources based on access, familiarity, or journal prestige—choices that can introduce citation bias without being inherently inaccurate.

Finally, quotation accuracy is not always captured by distinguishing between correct or erroneous quotations—subtle differences in meanings may not fit well in such a coarse dichotomy. In fact, the idea of „truth “ itself is complex [66] and, on an epistemological level, has been a „topic of perennial interest to philosophers “ (Michael Lynch [66]). However, on a pragmatic level, for the purposes of this study in the field of medicine, it is reassuring how often it is possible indeed to subdivide quotations into correct and inaccurate ones. Of note, a quotation can be correct but if the referenced work itself is not valid, the quoted fact is compromised.

### Interpretation

It is sobering that quotation inaccuracy is at a relatively high level and has not improved since it has been widely publicized forty years ago. Obviously, an integral part of scientific conduct is prone to error. However, while it is plausible that wrong quotations may be detrimental to science, there is little firm evidence on the consequences of quotation inaccuracy. Baethge (2020) reported that 21% of wrong quotations were of high, and 74% of wrong quotations of intermediate importance for the message of the paper, and only few were negligible (5%).

Quotation errors are not necessarily the result of poor scholarly work. In the authors’ experience from writing, reviewing and editing, quotation errors, especially secondary quotations and minor errors, can happen easily, particularly if authors feel under time pressure. Even major errors may creep into a manuscript without ill intent, for example, as a transmission failure. Another benign reason for error is simple misinterpretation of a source. It is also important to note that a paper or a claim is not automatically invalidated because quotations turn out to be wrong.

Several remedies (Table 2) have been repeatedly recommended but the situation has not improved. It may be time to change measures, for example, to ask authors for a written statement attesting the integrity of the quotations [67]. An entirely new factor is artificial intelligence (AI): As Peoples and co-authors state [67], AI may be a cause as well as a cure for quotation inaccuracy. Relying uncritically on AI in literature search and writing may increase quotation errors but, at the same time, AI may help in detecting errors. To the extent that AI is dependent on the written record, it may simply propagate quotation errors. For now, while we believe it is advisable to remain sceptical, it is impossible to predict AI’s effect on quotations, but it may be that the present estimate of quotation errors is among the last largely unafflicted by AI.

**Table 2:** Recommendations for avoiding quotation errors

Recommendations for authors:
o Read the quoted paper
o Do not quote original work from reviews (secondary quotation)
o Do not quote from abstracts of original articles
o Place references directly to the claim, not at the end of a sentence or a paragraph
o Do not create chain references unless absolutely necessary
Recommendations for editors:
o Communicate to authors that accuracy in quotations is expected and will be checked
o Carry out spot checks of quotations
o Consider adopting Harvard referencing style instead of Vancouver referencing style
o Consider using annotations or footnotes rather than reference lists only
o Consider using the help of AI in checking references
o Consider asking authors for a statement that quotations are correct (i.e., “works cited statement”)
Recommendations for reviewers:
o Carry out spot checks of quotations
o Consider using the help of AI in checking references
Recommendations for readers:
o Be aware that quotations may be wrong
o Carry out spot checks of quotations

Readers should not rely on journal prestige in estimating the correctness of quotations—we found only a weak negative correlation with journal impact factors explaining merely 7.5% of the variance. Also, other possible predictors, evaluated in our study, such as type of specialty and number of references, provide no reliable guidance.

In research, future investigators should employ standardized assessments: Distinguishing total, major, and minor quotation errors has established itself as a categorization with sufficient reliability and high face validity. We also recommend secondary quotations as a category. Researchers should check all quotations to a reference in a paper – at least they should provide the denominator of quotations actually investigated. We also argue to allow for more than one error per quotation. Finally, two raters should independently look into quotations.

## Conclusion

In conclusion, we found that one in every 6 quotations is incorrect, half of them in a major way and without improvement during the last 40 years. The field may need to develop new measures to improve quotations – a core element of scientific work.

## Supplementary Information

Supplementary Material 1.

## Data Availability

Data is available from the corresponding author (CB) upon reasonable request.

## Abbreviations

PRISMA: Preferred Reporting Items for Systematic Reviews and Meta-Analyses
OSF: Open Science Framework
JBI: Joanna Briggs Institute
RoB: Risk of Bias
CI: Confidence Interval
SD: Standard Deviation
