# Supplementary material for: Systematic review and meta-analysis of quotation inaccuracy in medicine
Source: Res Integr Peer Rev. 2025 Jul 23;10:13. doi: 10.1186/s41073-025-00173-z (PMC12285159; doi:10.1186/s41073-025-00173-z)

Supplemental Material

eTables & eFigures

eTable 1. Prisma checklist

eTable 2 Search Terms

eTable 3. Risk of bias tool: Adjusted JCI

eTable 4 Study Characteristics

eTable 5. Sensitivity analysis: Re-calculation of main analysis using arcsine square root data transformation and

eTable 6: Potential Risks and Opportunities of AI Tools in Scientific Referencing

Sidik & Jonkman tau^2^ estimation

eFigure 1. PRISMA-Flowchart

eFigure 2. Forest plot major quotation errors

eFigure 3. Forest plot minor quotation errors.

eFigure 4. Forest plot secondary quotation errors

eFigure 5. Meta-regression of time of observation on major quotation errors.

eFigure 6. Meta-regression of time of observation on minor quotation errors.

eFigure 7. Meta-regression of time of observation on secondary quotation errors

eFigure 8. Funnel plot total quotation errors

| **Section and Topic** | **Item #** | **Checklist item** | **Location where item is reported** |
| --- | --- | --- | --- |
| **TITLE** | | |  |
| Title | 1 | Identify the report as a systematic review. | p. 1 |
| **ABSTRACT** | | |  |
| Abstract | 2 | See the PRISMA 2020 for Abstracts checklist. | p. 2 |
| **INTRODUCTION** | | |  |
| Rationale | 3 | Describe the rationale for the review in the context of existing knowledge. | p. 3-4 |
| Objectives | 4 | Provide an explicit statement of the objective(s) or question(s) the review addresses. | p. 3-4 |
| **METHODS** | | |  |
| Eligibility criteria | 5 | Specify the inclusion and exclusion criteria for the review and how studies were grouped for the syntheses. | p. 5 |
| Information sources | 6 | Specify all databases, registers, websites, organisations, reference lists and other sources searched or consulted to identify studies. Specify the date when each source was last searched or consulted. | p. 6 |
| Search strategy | 7 | Present the full search strategies for all databases, registers and websites, including any filters and limits used. | eTable 2 (cited on page 6) |
| Selection process | 8 | Specify the methods used to decide whether a study met the inclusion criteria of the review, including how many reviewers screened each record and each report retrieved, whether they worked independently, and if applicable, details of automation tools used in the process. | p. 6 |
| Data collection process | 9 | Specify the methods used to collect data from reports, including how many reviewers collected data from each report, whether they worked independently, any processes for obtaining or confirming data from study investigators, and if applicable, details of automation tools used in the process. | p. 7 |
| Data items | 10a | List and define all outcomes for which data were sought. Specify whether all results that were compatible with each outcome domain in each study were sought (e.g. for all measures, time points, analyses), and if not, the methods used to decide which results to collect. | p. 7-8 |
|  | 10b | List and define all other variables for which data were sought (e.g. participant and intervention characteristics, funding sources). Describe any assumptions made about any missing or unclear information. | p. 8 |
| Study risk of bias assessment | 11 | Specify the methods used to assess risk of bias in the included studies, including details of the tool(s) used, how many reviewers assessed each study and whether they worked independently, and if applicable, details of automation tools used in the process. | p. 9 |
| Effect measures | 12 | Specify for each outcome the effect measure(s) (e.g. risk ratio, mean difference) used in the synthesis or presentation of results. | p. 10 |
| Synthesis methods | 13a | Describe the processes used to decide which studies were eligible for each synthesis (e.g. tabulating the study intervention characteristics and comparing against the planned groups for each synthesis (item #5)). | p. 10-12 |
|  | 13b | Describe any methods required to prepare the data for presentation or synthesis, such as handling of missing summary statistics, or data conversions. | p. 10-12 |
|  | 13c | Describe any methods used to tabulate or visually display results of individual studies and syntheses. | p. 10-12 |
|  | 13d | Describe any methods used to synthesize results and provide a rationale for the choice(s). If meta-analysis was performed, describe the model(s), method(s) to identify the presence and extent of statistical heterogeneity, and software package(s) used. | p. 10-12 |
|  | 13e | Describe any methods used to explore possible causes of heterogeneity among study results (e.g. subgroup analysis, meta-regression). | p. 10-12 |
|  | 13f | Describe any sensitivity analyses conducted to assess robustness of the synthesized results. | p. 10-12 |
| Reporting bias assessment | 14 | Describe any methods used to assess risk of bias due to missing results in a synthesis (arising from reporting biases). | p. 14 |
| Certainty assessment | 15 | Describe any methods used to assess certainty (or confidence) in the body of evidence for an outcome. | p. 14 |
| **RESULTS** | | |  |
| Study selection | 16a | Describe the results of the search and selection process, from the number of records identified in the search to the number of studies included in the review, ideally using a flow diagram. | p. 15 |
|  | 16b | Cite studies that might appear to meet the inclusion criteria, but which were excluded, and explain why they were excluded. | p. 15 |
| Study characteristics | 17 | Cite each included study and present its characteristics. | eTable 4 (referenced on p. 16) |
| Risk of bias in studies | 18 | Present assessments of risk of bias for each included study. | p. 18 |
| Results of individual studies | 19 | For all outcomes, present, for each study: (a) summary statistics for each group (where appropriate) and (b) an effect estimate and its precision (e.g. confidence/credible interval), ideally using structured tables or plots. | p. 17-18 |
| Results of syntheses | 20a | For each synthesis, briefly summarise the characteristics and risk of bias among contributing studies. | p. 16-18 |
|  | 20b | Present results of all statistical syntheses conducted. If meta-analysis was done, present for each the summary estimate and its precision (e.g. confidence/credible interval) and measures of statistical heterogeneity. If comparing groups, describe the direction of the effect. | p. 17 |
|  | 20c | Present results of all investigations of possible causes of heterogeneity among study results. | p. 18 |
|  | 20d | Present results of all sensitivity analyses conducted to assess the robustness of the synthesized results. | p. 19 |
| Reporting biases | 21 | Present assessments of risk of bias due to missing results (arising from reporting biases) for each synthesis assessed. | p. 19 |
| Certainty of evidence | 22 | Present assessments of certainty (or confidence) in the body of evidence for each outcome assessed. | p. 20 |
| **DISCUSSION** | | |  |
| Discussion | 23a | Provide a general interpretation of the results in the context of other evidence. | p. 21 |
|  | 23b | Discuss any limitations of the evidence included in the review. | p. 22 |
|  | 23c | Discuss any limitations of the review processes used. | p. 22 |
|  | 23d | Discuss implications of the results for practice, policy, and future research. | p. 23-24 |
| **OTHER INFORMATION** | | |  |
| Registration and protocol | 24a | Provide registration information for the review, including register name and registration number, or state that the review was not registered. | p. 5 (OSF) |
|  | 24b | Indicate where the review protocol can be accessed, or state that a protocol was not prepared. | p. 5 |
|  | 24c | Describe and explain any amendments to information provided at registration or in the protocol. | p. 14 |
| Support | 25 | Describe sources of financial or non-financial support for the review, and the role of the funders or sponsors in the review. | p. 24 |
| Competing interests | 26 | Declare any competing interests of review authors. | p. 24 |
| Availability of data, code and other materials | 27 | Report which of the following are publicly available and where they can be found: template data collection forms; data extracted from included studies; data used for all analyses; analytic code; any other materials used in the review. | p. 24 |

*From:*  Page MJ, McKenzie JE, Bossuyt PM, Boutron I, Hoffmann TC, Mulrow CD, et al. The PRISMA 2020 statement: an updated guideline for reporting systematic reviews. BMJ 2021;372:n71. doi: 10.1136/bmj.n71. This work is licensed under CC BY 4.0. To view a copy of this license, visit <https://creativecommons.org/licenses/by/4.0/>

**eTable 2 Search Terms**

The literature search was conducted across two distinct databases: Medline (via PubMed) and the Web of Science Core Collection. Given that each database necessitates unique query syntax for systematically retrieving indexed literature, the following search strategies were developed accordingly:

*Pubmed:*

(accura* OR inaccura* OR error OR mistake OR correct* OR incorrect*) AND

(citation* OR quotation* OR reference* OR source OR bibliography) AND

(bibliography as topic[MeSH] OR periodicals as topic[MeSH])

*Web of Science:*

(ALL = accura* OR ALL = inaccura* OR ALL = error OR ALL = mistake OR ALL = correct* OR ALL = incorrect*) AND (ALL = citation* OR ALL = quotation* OR ALL = reference* OR ALL = source OR ALL = bibliography) AND (TS=bibliography OR TS=periodicals )

**eTable 3. Risk of bias tool: Adjusted JCI**

All additions by the authors are italicized

*The checklist consists of nine items on the appropriateness of key methodological aspects: (1) sample frame, (2) recruitment of study participants, (3) sample size, (4) description of subjects and setting, (5) sufficient coverage of the sample, (6) methods for case detection, (7) standardized measurement, (8) statistical analysis, and (9) response rate. We adjusted all items for RoB assessment of studies on quotation inaccuracy, e.g. , by including item definitions for random selection of quotations (revised item 2) or the use of two independent raters in determining quotation errors (revised item 7).*

JBI Critical Appraisal Checklist for
studies reporting prevalence data

Reviewer______________________________________ Date___________________________

Author________________________________________ Year_________ Record Number__________

|  | Yes | No | Unclear | Not applicable |
| --- | --- | --- | --- | --- |
| 1. Was the sample frame appropriate to address the target population? | □ | □ | □ | □ |
| 1. Were study participants sampled in an appropriate way? | □ | □ | □ | □ |
| 1. Was the sample size adequate? | □ | □ | □ | □ |
| 1. Were the study subjects and the setting described in detail? | □ | □ | □ | □ |
| 1. Was the data analysis conducted with sufficient coverage of the identified sample? | □ | □ | □ | □ |
| 1. Were valid methods used for the identification of the condition? | □ | □ | □ | □ |
| 1. Was the condition measured in a standard, reliable way for all participants? | □ | □ | □ | □ |
| 1. Was there appropriate statistical analysis? | □ | □ | □ | □ |
| 1. Was the response rate adequate, and if not, was the low response rate managed appropriately? | □ | □ | □ | □ |

Overall appraisal: Include □ Exclude □ Seek further info □

Comments (Including reason for exclusion)

__________________________________________________________________________________________________________________________________________________________________________________________________________________________________

JBI Critical Appraisal Checklist for Studies Reporting Prevalence Data

Answers: Yes, No, Unclear or Not/Applicable

1. **Was the sample frame appropriate to address the target population?**

This question relies upon knowledge of the broader characteristics of the population of interest and the geographical area. If the study is of women with breast cancer, knowledge of at least the characteristics, demographics and medical history is needed. The term “target population” should not be taken to infer every individual from everywhere or with similar disease or exposure characteristics. Instead, give consideration to specific population characteristics in the study, including age range, gender, morbidities, medications, and other potentially influential factors. For example, a sample frame may not be appropriate to address the target population if a certain group has been used (such as those working for one organisation, or one profession) and the results then inferred to the target population (i.e. working adults). A sample frame may be appropriate when it includes almost all the members of the target population (i.e. a census, or a complete list of participants or complete registry data).

*Jergas & Baethge:*

*To rate “yes” a study needs to have sampled quotations from a range of sources, for example, two or several journals representative of a field. In the event that authors select top journals in a field, e.g., defined by Impact Factor, we consider this as sufficiently representative of a field (giving the field, so to say, the benefit of the doubt, but also because earlier studies have shown no clear association of impact factor and quotation accuracy).*

*If quotations to only a single study are sampled, we consider it a “no” because, for the purposes of our study, it is impossible to conclude from this sample to a wider population. While it may be possible that authors of such studies themselves do not generalize their findings, although in our experience this is rare, we cannot draw conclusions beyond these highly selected quotations. There also exists the possibility of selection bias because often quotations to single papers are selected based on the experience of inaccurate conclusion to particular papers.*

1. **Were study participants recruited in an appropriate way?**

Studies may report random sampling from a population, and the methods section should report how sampling was performed. Random probabilistic sampling from a defined subset of the population (sample frame) should be employed in most cases, however, random probabilistic sampling is not needed when everyone in the sampling frame will be included/ analysed. For example, reporting on all the data from a good census is appropriate as a good census will identify everybody. When using cluster sampling, such as a random sample of villages within a region, the methods need to be clearly stated as the precision of the final prevalence estimate incorporates the clustering effect. Convenience samples, such as a street survey or interviewing lots of people at a public gatherings are not considered to provide a representative sample of the base population.

*Jergas & Baethge:*

*To rate “yes”, there has to be a random element in the selection of quotations.*

*Studies in which quotations were selected on a non-random basis receive a “no” rating. This includes studies mentioned under 1 that have investigated quotations to single works even though they may sometimes have double-checked all quotations. Again, the sampling may be appropriate for the main objective of a given study but for the purposes of our study there can be no representative sampling from a particularly non-representative population.*

1. **Was the sample size adequate?**

The larger the sample, the narrower will be the confidence interval around the prevalence estimate, making the results more precise. An adequate sample size is important to ensure good precision of the final estimate. Ideally we are looking for evidence that the authors conducted a sample size calculation to determine an adequate sample size. This will estimate how many subjects are needed to produce a reliable estimate of the measure(s) of interest. For conditions with a low prevalence, a larger sample size is needed. Also consider sample sizes for subgroup (or characteristics) analyses, and whether these are appropriate. Sometimes, the study will be large enough (as in large national surveys) whereby a sample size calculation is not required. In these cases, sample size can be considered adequate.

When there is no sample size calculation and it is not a large national survey, the reviewers may consider conducting their own sample size analysis using the following formula: (Naing et al. 2006, Daniel 1999)

n= Z2P(1-P)

d2

Where:

n= sample size

Z = Z statistic for a level of confidence

P = Expected prevalence or proportion (in proportion of one; if 20%, P = 0.2)

d = precision (in proportion of one; if 5%, d=0.05)

**Ref:**

Naing L, Winn T, Rusli BN. Practical issues in calculating the sample size for prevalence studies Archives of Orofacial Sciences. 2006;1:9-14.

Daniel WW. Biostatistics: A Foundation for Analysis in the Health Sciences.

Edition. 7th ed. New York: John Wiley & Sons. 1999.

*Jergas & Baethge:*

*For a “yes” rating there should have been a sample-size calculation or, if the sample has been randomly selected, a sample size of at least 200 quotations. This figure is somewhat arbitrary and is a compromise between a sample size that allows for some degree of statistical accuracy, for example, in producing a 95%-confidence interval of about 7% around 50%, and the fact that in meta-analyses underpowered studies, with all their inherent statistical difficulties, can be meaningfully added with other studies to arrive at a robust summary estimate. In fact, while an underpowered study may be very difficult to interpret as a single study, it may proof valuable as part of a meta-analysis.*

1. **Were the study subjects and setting described in detail?**

Certain diseases or conditions vary in prevalence across different geographic regions and populations (e.g. Women vs. Men, sociodemographic variables between countries). The study sample should be described in sufficient detail so that other researchers can determine if it is comparable to the population of interest to them.

*Jergas & Baethge:*

*A “yes” rating is conditional on a description of the specialty, the sources (e.g., which journals and articles), the time of coverage, and the quotations chosen (including the definitions of numerators and denominators).*

1. **Was data analysis conducted with sufficient coverage of the identified sample?**

Coverage bias can occur when not all subgroups of the identified sample respond at the same rate. For instance, you may have a very high response rate overall for your study, but the response rate for a certain subgroup (i.e. older adults) may be quite low.

*Jergas & Baethge:*

*Certain article types study authors aimed for, e.g., original papers or review articles, were not found or underrepresented. Otherwise, this item will receive a “yes”.*

1. **Were valid methods used for the identification of the condition?**

Here we are looking for measurement or classification bias. Many health problems are not easily diagnosed or defined and some measures may not be capable of including or excluding appropriate levels or stages of the health problem. If the outcomes were assessed based on existing definitions or diagnostic criteria, then the answer to this question is likely to be yes. If the outcomes were assessed using observer reported, or self-reported scales, the risk of over- or under-reporting is increased, and objectivity is compromised. Importantly, determine if the measurement tools used were validated instruments as this has a significant impact on outcome assessment validity.

*Jergas & Baethge:*

*In our study, this item refers to the definition of quotation errors. If a study contains a definition that is in line with the literature, as exemplified in Jergas & Baethge (2015), Mogull (2017), or Baethge (2020), the rating is “yes”. If the definition of a quotation error is out of touch with this line of research, the study is rated as “unclear” or even gets a “no”.*

1. **Was the condition measured in a standard, reliable way for all participants?**

Considerable judgment is required to determine the presence of some health outcomes. Having established the validity of the outcome measurement instrument (see item 6 of this scale), it is important to establish how the measurement was conducted. Were those involved in collecting data trained or educated in the use of the instrument/s? If there was more than one data collector, were they similar in terms of level of education, clinical or research experience, or level of responsibility in the piece of research being appraised? When there was more than one observer or collector, was there comparison of results from across the observers? Was the condition measured in the same way for all participants?

*Jergas & Baethge:*

*The core condition for a “yes” in this domain is that at least two raters independently evaluated the quotations under study.*

1. **Was there appropriate statistical analysis?**

Importantly, the numerator and denominator should be clearly reported, and percentages should be given with confidence intervals. The methods section should be detailed enough for reviewers to identify the analytical technique used and how specific variables were measured. Additionally, it is also important to assess the appropriateness of the analytical strategy in terms of the assumptions associated with the approach as differing methods of analysis are based on differing assumptions about the data and how it will respond.

*Jergas & Baethge:*

*A study will receive a “yes” rating if numerators and denominators are clearly reported. Based on those figures, confidence intervals can be calculated and thus the presentation of confidence intervals is not necessary for a “yes”.*

1. **Was the response rate adequate, and if not, was the low response rate managed appropriately?**

A large number of dropouts, refusals or “not founds” amongst selected subjects may diminish a study’s validity, as can a low response rates for survey studies. The authors should clearly discuss the response rate and any reasons for non-response and compare persons in the study to those not in the study, particularly with regards to their socio-demographic characteristics. If reasons for non-response appear to be unrelated to the outcome measured and the characteristics of non-responders are comparable to those who do respond in the study (addressed in question 5, coverage bias), the researchers may be able to justify a more modest response rate.

*Jergas & Baethge:*

*This item is applicable to our study only as some quoted sources were not retrieved in a study, for example, because a source is not among the holdings of a library or a library system. If lack of retrieval remains below 10% we rate “yes”, otherwise “no”.*

**eTable 4 Study Characteristics**

| **First**  **Author/**  **Year** | **Observation Period** | **Country**  **of Origin** | **Study**  **Size** | **Imputed**  **denominator** | **Risk of Bias** | **Predictors: Impact Factor & References** | **Study Type** | **Medical**  **Specialty** | **Independent**  **Raters** |
| --- | --- | --- | --- | --- | --- | --- | --- | --- | --- |
| **Armstrong (2018)** | 2017 | USA | 400 | 624.0 | 9 | Impact factor | Medical journal articles | ENT | Yes |
| **Baethge (2020)** | 2010/  2011 | Germany | 235 | NA | 8 | Impact factor | Medical journal articles | Psychiatry | Yes |
| **Choi (2021)** | 2003 to 2017 | USA | 1200 | NA | 9 | Number of citations | Medical journal articles | Surgery | Yes |
| **Cimen (2016)** | 2015 | Turkey | 438 | 753.3 | 4 | Number of references | Medical journal articles | Urology | Yes |
| **Gazendam (2021)** | 2019 | Canada | 250 | NA | 8 | Number of references | Medical journal articles | Sports Medicine | Unclear |
| **Hinchcliff (1993)** | 1990 | USA | 102 | NA | 6 | NA | Medical journal articles but no book reviews, letters, legal briefs, editorials, news articles and tutorials | Veterinary Medicine | No |
| **Homeier (2024)** | Dec 2021 and Jan 2022 | USA | 769 references, 1082 citations | NA | 8 | Impact factor Number of references, | Medical journal articles but no Editorials, editorial commentaries, and letters to the editor | Sports Medicine | Yes |
| **Hongmei (2018)** | 2014 | China | 147 (Chinese Series), 155 (Journal Series), Total 302 (Chinese); 567 (Chinese Series), 633 (Journal Series), Total 1200 (English) | NA | 8 | NA | Medical journal articles | General Medicine | Unclear |
| **Hui (2020)** | 2013 to  2018 | UK | 236 | NA | 6 | NA | Policy documents | Psychiatry | No |
| **Ju (2004)** | 2004 | China | 1456 (1184 in foreign language, 272 in Chinese) | 1831.4 | 7 | NA | Medical journal articles | General Medicine | Unclear |
| **Montenegro a (2021)** | 2019 | USA | 240 | 374.4 | 8 | Number of references | Medical journal articles, but not those included in or written for supplements, editorials, letters and correspondence | Neurosurgery | Yes |
| **Montenegro b (2021)** | 2019 | USA | 220 | 343.2 | 8 | Number of references | Medical journal articles, but not those included in or written for supplements, editorials, letters and correspondence | Neurosurgery (Spine Surgery) | Yes |
| **Moran (2022)** | 2014 - 2020 (2017) | Spain | 147 | NA | 4 | NA | References to sytematic review protocols and meta-analyses | Complimentary and Alternative Medicine, CAM | Yes |
| **Pavlovic (2021)** | A: to 2017 B: to 2019 | Serbia/USA/Germany | 7438 (total), 2526 (feasibility set), 4912 (Verification set) | NA | 7 | Impact factor, number of references | Medical journal articles including original articles, review, comments, letters, commincation, case reports, study protocols, guidelines, pilot studies, opinion articles, editorial comments | General Medicine | Yes |
| **Sauder (2022)** | 2015 to 2020 | USA | 5973 | NA | 7 | NA | Original research articles | Surgery | Unclear |
| **Stather (2014)** | 2012/ 2013 | United Kingdom | 43 | NA | 4 | NA | Medical journal articles | Surgery | No |
| **Trost (2014)** | 2013 | USA | 468 | NA | 5 | NA | Original research articles and meta-analyses | Urology | Unclear |
| **Al-Benna**  **(2009)** | 2006 | Germany/UK | 113 | 176.3 | 7 | Number of references | Original research articles | Surgery | No |
| **Awrey**  **(2010)** | 2007 | USA | 900 | 1404.0 | 9 | Impact Factor | Original research articles | Surgery | Yes |
| **Buchan**  **(2003)** | 2003 | USA/UK | 200 | NA | 9 | NA | Medical journal articles | Ophthalmology | Yes |
| **Bujize**  **(2011)** | 2000 to 2009 | USA/Canada/Netherlands | 2011 | NA | 9 | Impact factor, number of references | Medical journal articles | Orthopedics | Yes |
| **Davids**  **(2010)** | 2007/2008 | Israel/USA | 200 | NA | 9 | Impact factor, number of references | Medical journal articles but no review articles or case reports | Orthopedics | Yes |
| **De Lacey**  **(1985)** | 1984 | New Zealand/UK | 300 | 435.2 | 7 | NA | Medical journal articles | General medicine | Yes |
| **Eichorn**  **(1987)** | 1986 | USA | 150 | 234.0 | 8 | NA | Original research articles and short communications | Public health | Yes |
| **Evans**  **(1990)** | 1987 | USA | 137 | 213.7 | 7 | NA | Medical journal articles | Surgery | Yes |
| **Fenton**  **(2000)** | 1997 | UK/Ireland | 153 | 236.7 | 7 | Impact factor | Medical journal articles | ENT | No |
| **George**  **(1994)** | 1992 | USA | 239 | NA | 8 | Number of references | Medical journal articles but no supplements, correspondence or editorials | Dermatology | Yes |
| **Goldberg**  **(1993)** | 1991 | USA | 145 | 226.2 | 8 | NA | Medical journal articles but no letters to the editor | Emergency medicine | Yes |
| **Gosling**  **(2004)** | 2000/2001 | Australia | 320 | NA | 8 | NA | Original research articles | Manual Therapy | No |
| **Gupta**  **(2005)** | 2002 | India | 176 | 274.6 | 4 | NA | Original research articles | Pediatrics | Yes |
| **Hansen**  **(1994)** | 1993 | USA | 95 | 148.2 | 5 | Number of references | Medical journal articles | Radiology | No |
| **Hobma**  **(1992)** | 1991 | Netherlands | 99 | 154.4 | 5 | Number of references | Medical journal articles | General Medicine | No |
| **Lawson**  **(1999)** | 1997 | United Kingdom | 147 | 229.3 | 7 | NA | Medical journal articles | Psychiatry | No |
| **Lee^1^**  **(1999)** | 1993 | South Korea | 200 | 312.0 | 6 | NA | Original research articles and short communications | Dermatology | No |
| **Lee^1^ (1995)** | 1993 | South Korea | 70 | 109.2 | 6 | NA | Original research articles and short communications | Dermatology | No |
| **Lowry**  **(1985)** | 1984 | UK | 61 | NA | 5 | NA | Letters | General Medicine | No |
| **Lukic**  **(2004)** | 2001 | Croatia/USA | 199 | NA | 7 | NA | Medical journal articles | Gross Anatomy | Yes |
| **Luo**  **(2013)** | 2009 | USA | 249 | NA | 9 | NA | Medical journal articles | Orthopedics | Yes |
| **Mertens**  **(2011)** | 2010 | Germany | 50 | NA | 6 | NA | Medical journal articles | General Medicine | Yes |
| **Neihouse**  **(1989)** | 1987 | USA | 99 | NA | 7 | NA | Review articles | Pharmacology | No |
| **Pieters**  **(2001)** | 1999 | Netherlands | 95 | 148.2 | 5 | NA | Medical journal articles | Psychiatry | Yes |
| **Puttermann**  **(1992)** | 1990 | Israel | 120 | NA | 6 | NA | Original research articles, review articles, editorials and special articles (e.g. public health or medical history) | General Medicine | No |
| **Reddy**  **(2008)** | 2004 | United Kingdom | 255 | 397.8 | 9 | Number of references | Medical journal articles but no reviews, case reports or letters | Surgery | Yes |
| **Schulmeister**  **(1998)** | 1995/1996 | USA | 180 | 280.8 | 8 | Number of references | Medical journal articles | Nursing | Yes |
| **Singh**  **(2009)** | 2008 | India | 46 | 71.8 | 5 | NA | Medical journal articles | Dermatology | No |
| **Warren**  **(1997)** | unclear | USA | 382 | 595.9 | 3 | NA | Medical journal articles | Infectious Diseases | No |

1: Given that both publications by Lee & Lee (1995 and 1999) draw on the same sample, we consider them methodologically equivalent for the purposes of this analysis.

**eTable 5. Sensitivity analysis: Re-calculation of main analysis using arcsine square root data transformation and Sidik & Jonkman tau^2^ estimation**

| **Meta-Analysis** | **Total** | **Major** | **Minor** | **Secondary** |
| --- | --- | --- | --- | --- |
|  |  |  |  |  |
| **DerSimonian & Laird (main analysis)** |  |  |  |  |
| **Logit transformation (main analysis)** | **16,9 [14,1-20.1]** | **8,0 [6,4-9,9]** | **7,8 [5,7-10,6]** | **5,3 [3,3-8,5]** |
| Arcsine square root transformation | 18,0 [15,4-20,8] | 8,4 [6,7-10,3] | 8,8 [6,7-11,2] | 6,3 [4,0-9,1] |
|  |  |  |  |  |
| Sidik & Jonkman |  |  |  |  |
| Logit transformation | 16,8 [13,5-20,9] | 7,9 [6,1-10,1] | 7,8 [5,7-10,6] | 5,3 [3,4-8,3] |
| Arcsine square root transformation | 18,3 [14,5-22,4] | 8,4 [6,4-10,7] | 8,8 [6,4-11,6] | 6,3 [3,7-9,5] |
|  |  |  |  |  |

**eTable 6: Potential Risks and Opportunities of AI Tools in Scientific Referencing**

| **Category** | **Aspect** | **Description** |
| --- | --- | --- |
| **Potential Risks** | Hallucinated References | AI may generate plausible but non-existent citations. |
|  | Loss of Source Control | AI may obscure the original source or misattribute claims, mirroring secondary quotation issues. |
|  | Bias and Misleading Citations | AI may reinforce citation bias by favoring well-cited sources, neglecting less prominent ones. |
|  | Dilution of Expertise | Overreliance on AI can reduce critical appraisal of sources, weakening scholarly rigor. |
| **Potential Contributions** | Reference Validation Tools | AI can support accuracy checking of citations for both editors and authors. |
|  | Streamlined Literature Reviews | AI can surface relevant recent literature for manual curation by researchers. |
|  | Verifiable References | AI can assist in ensuring that claims are clearly linked to traceable, valid primary sources. |
|  | Bias Detection | AI tools may help identify citation imbalances (e.g., geographic or authorial bias). |

**eFigure 1. PRISMA-Flowchart**


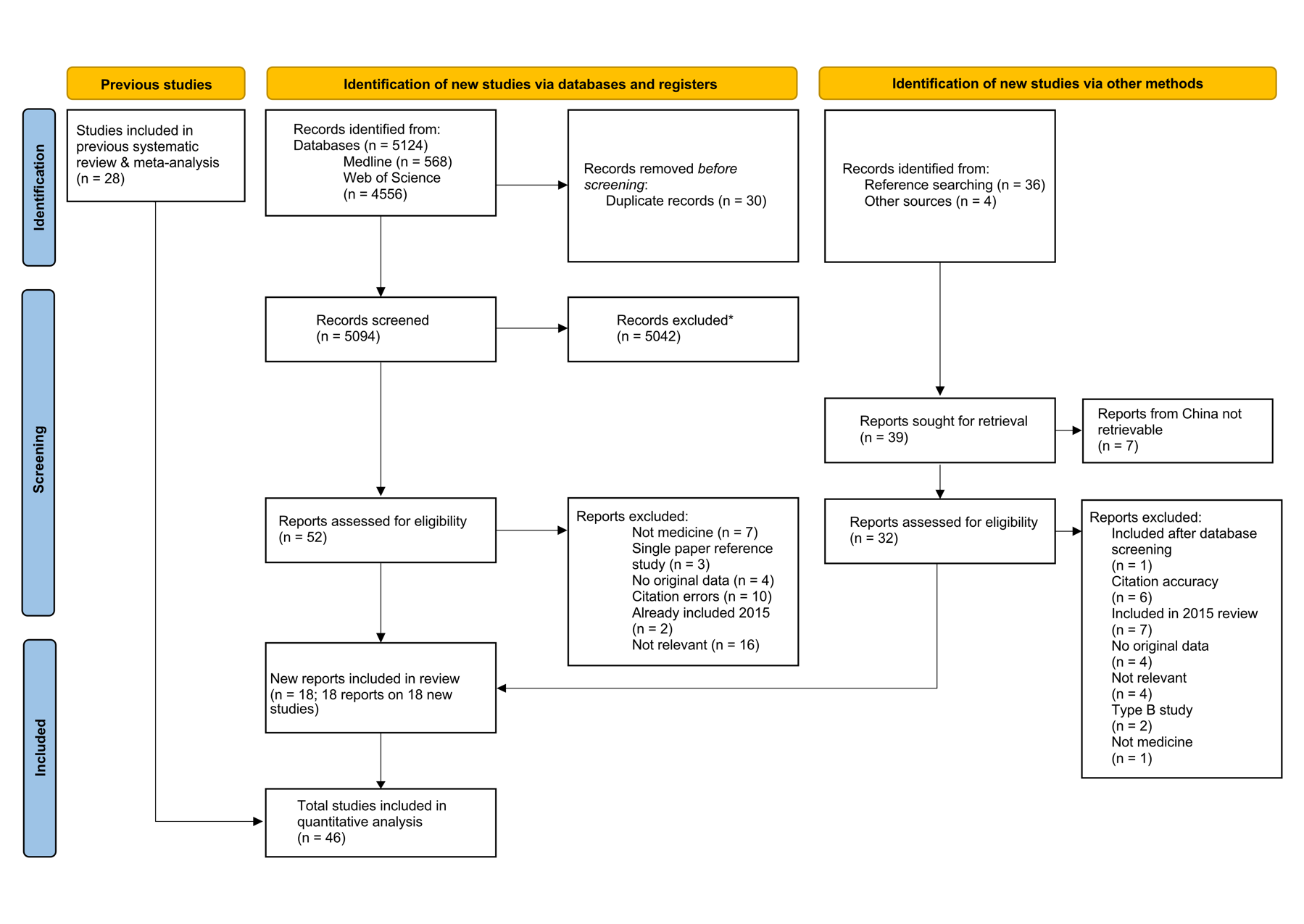


**eFigure 2. Forest plot major quotation errors**


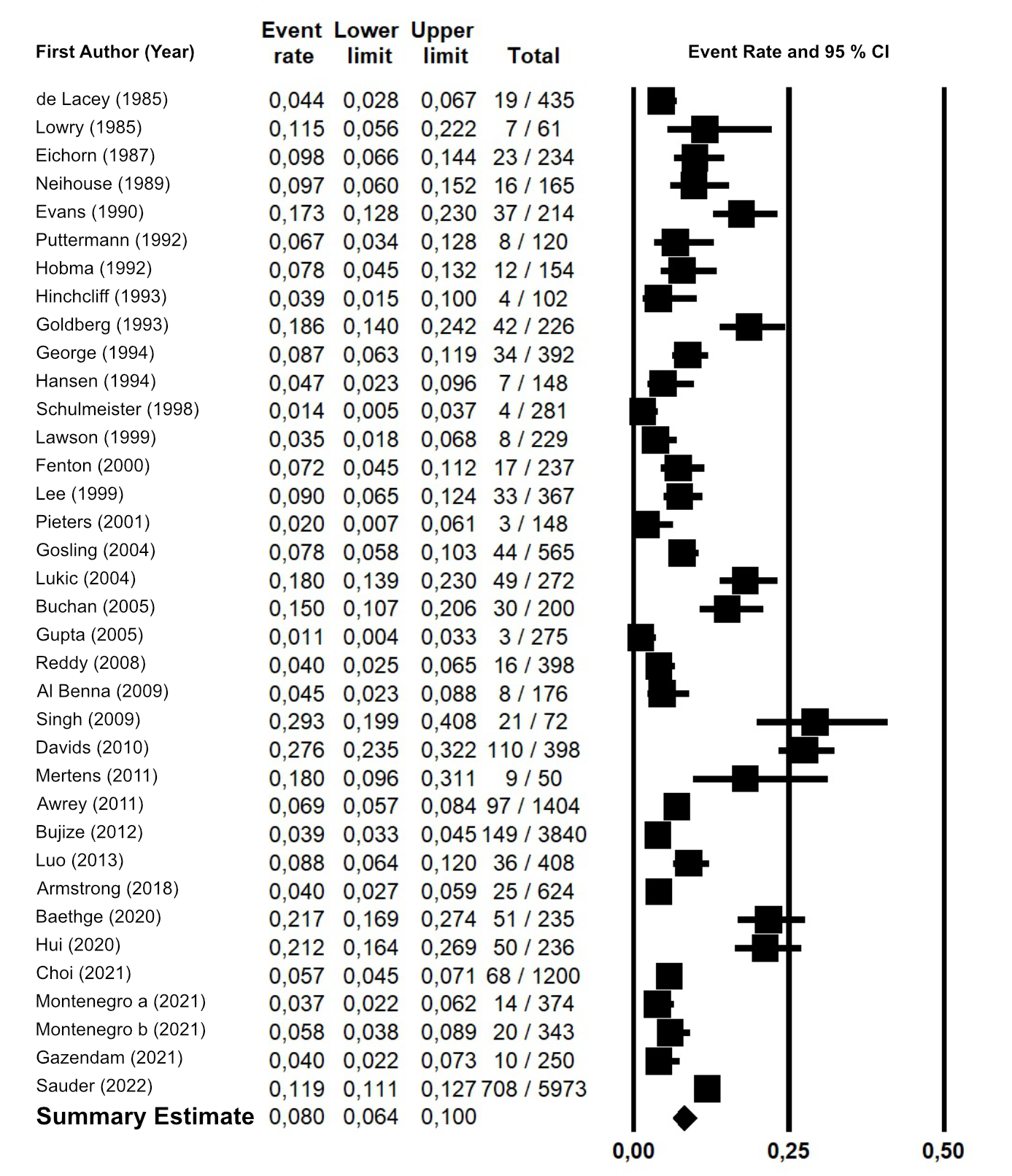


**eFigure 3. Forest plot minor quotation errors.**


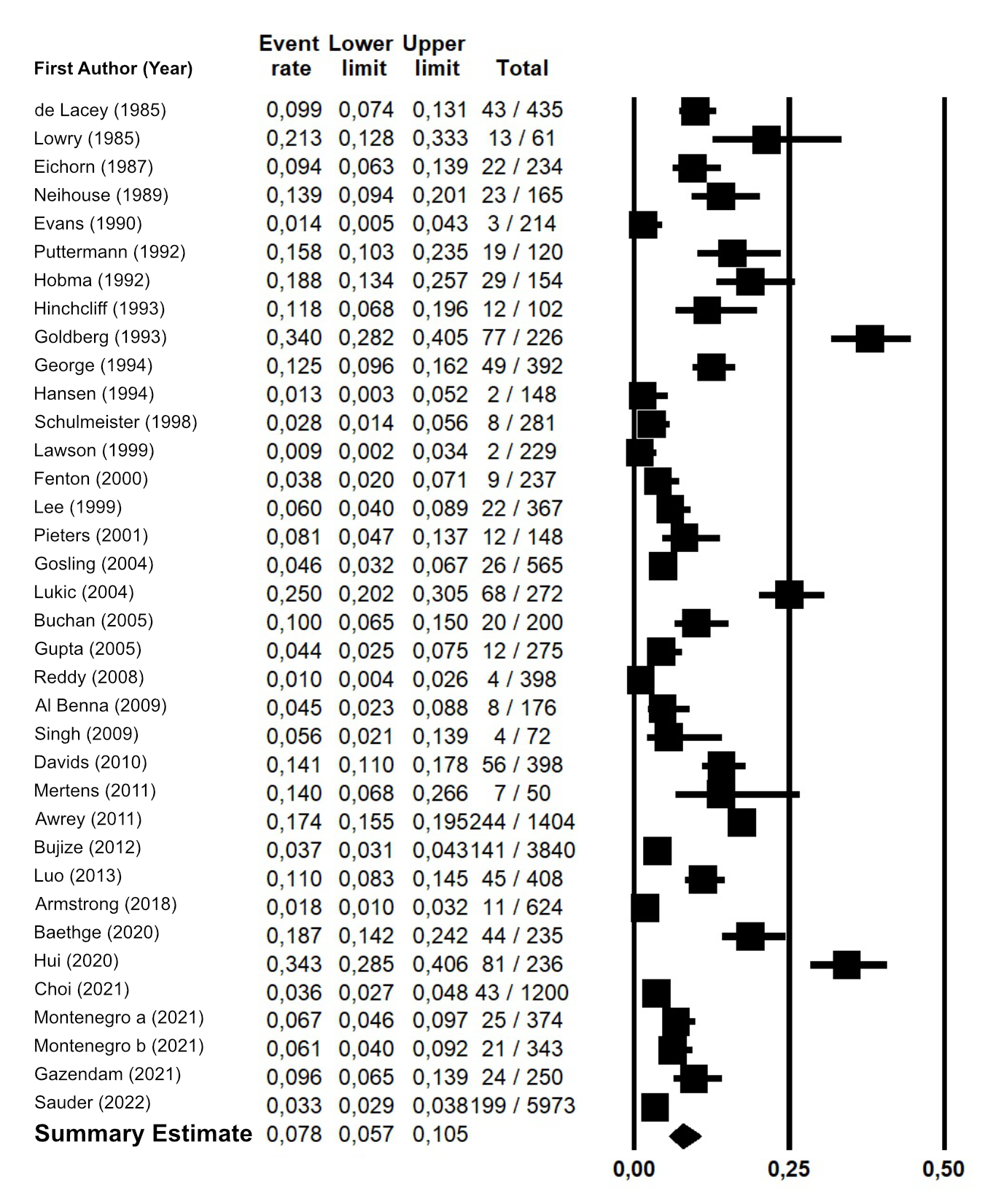


**eFigure 4. Forest plot secondary quotation errors**


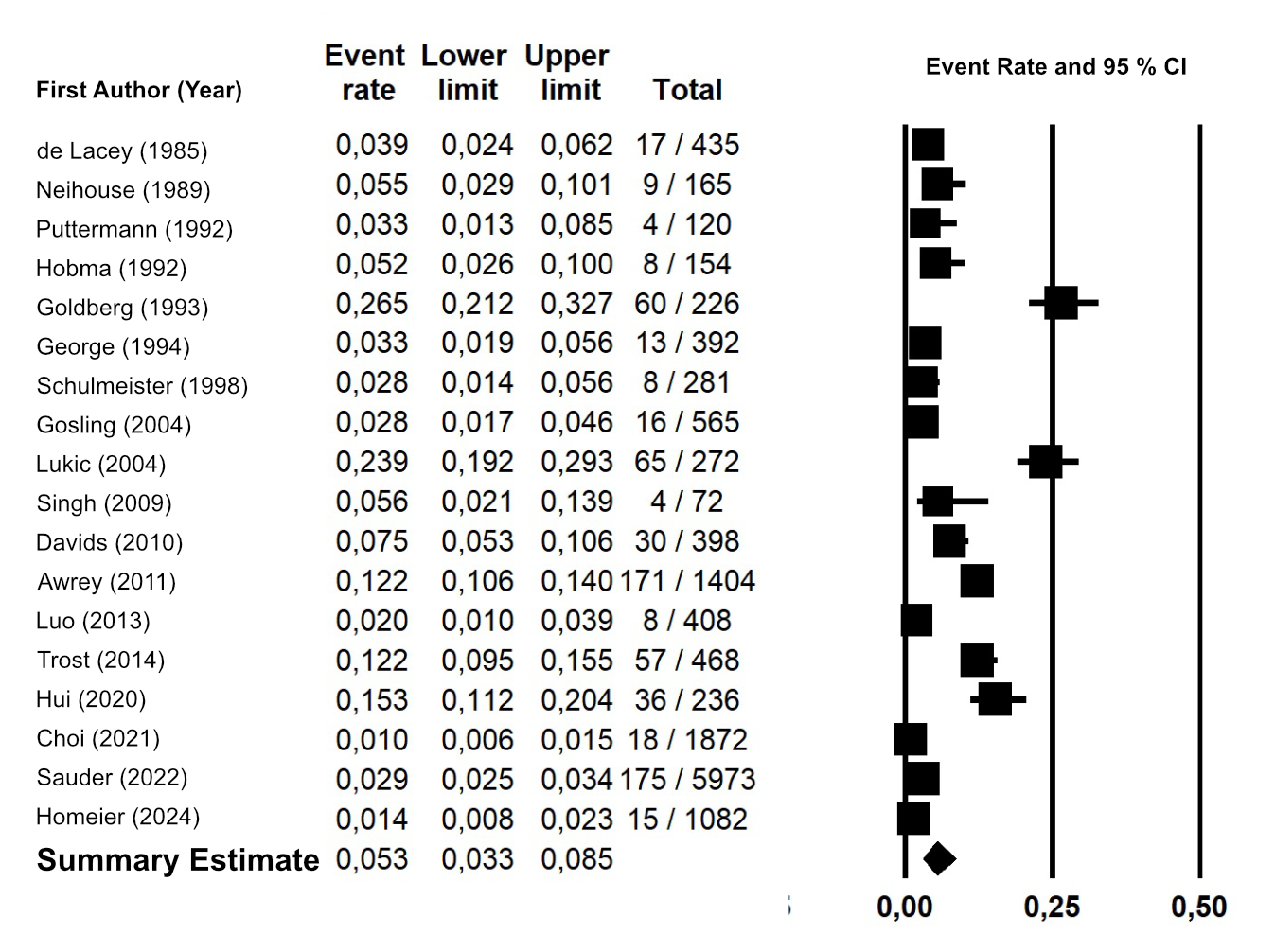


**eFigure 5. Meta-regression of time of observation on major quotation errors.**


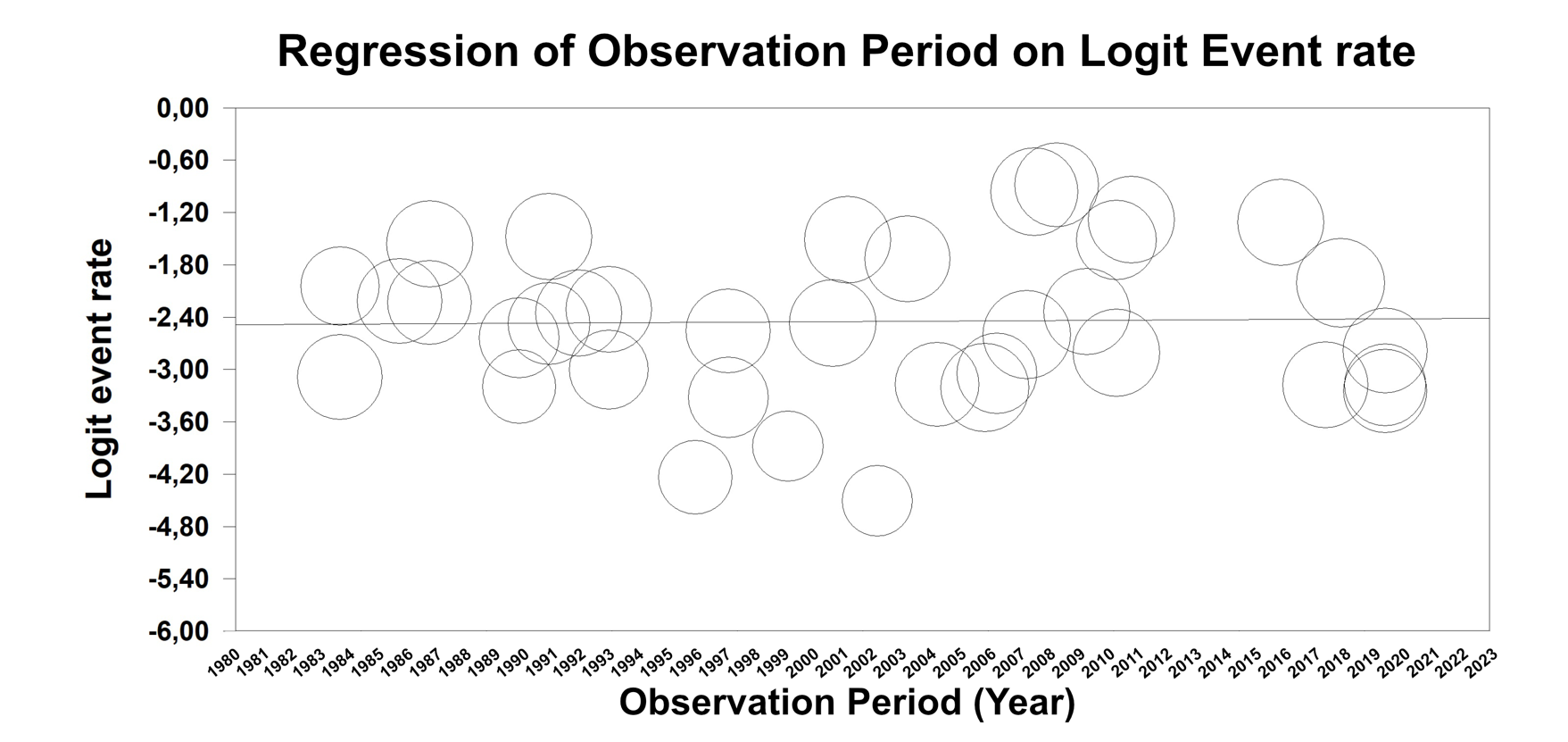


**eFigure 6. Meta-regression of time of observation on minor quotation errors.**


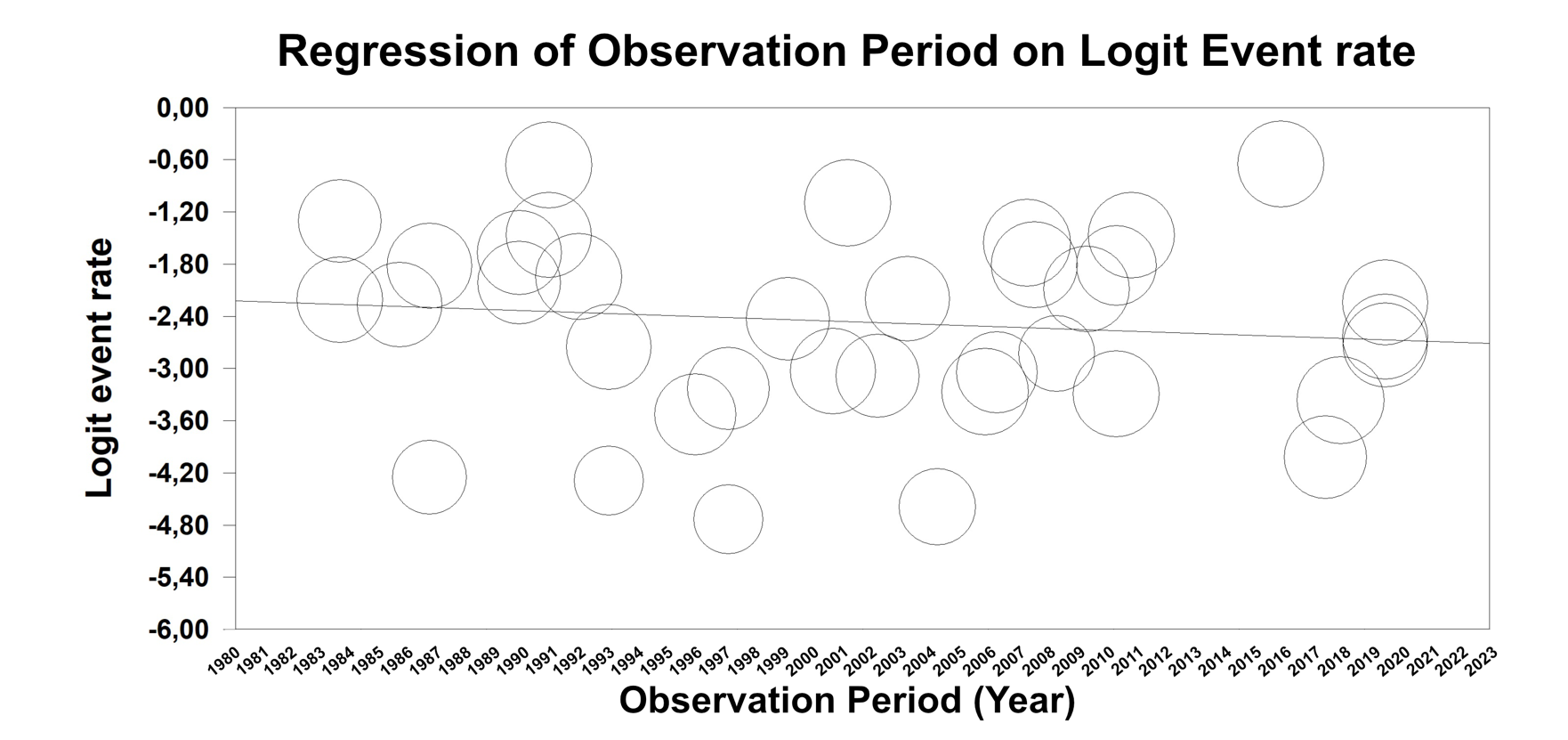


**eFigure 7. Meta-regression of time of observation on secondary quotation errors**


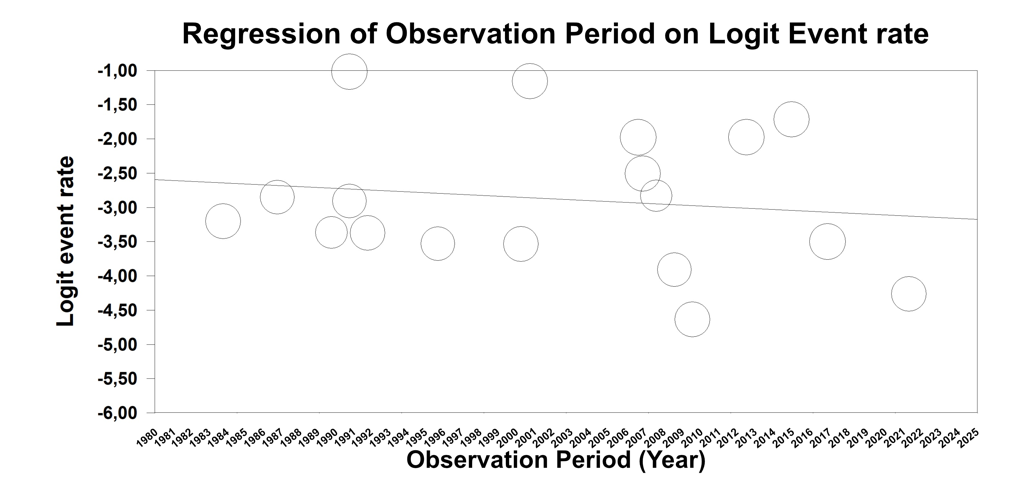


**eFigure 8. Funnel plot total quotation errors**


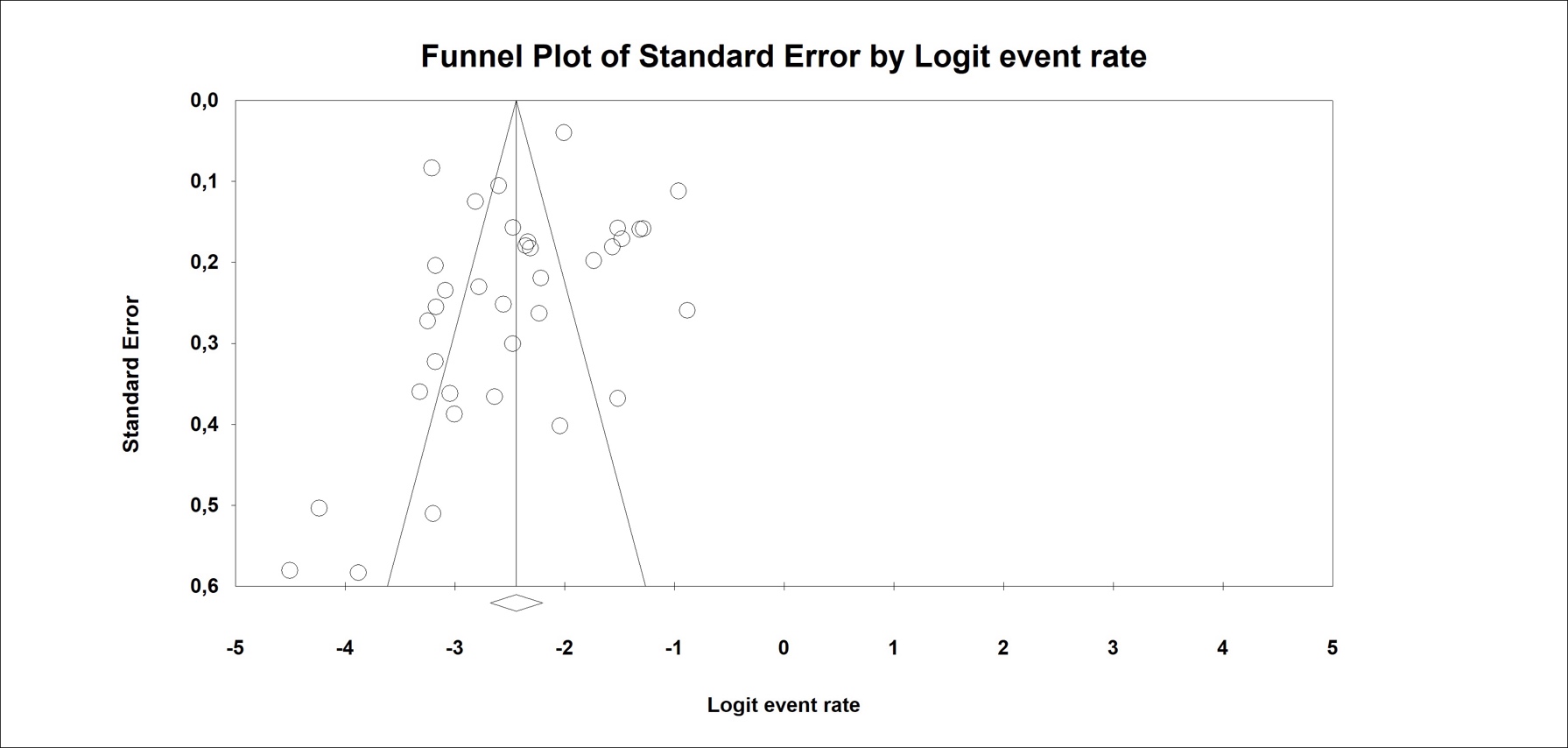

Supplement: Supplementary file 1 — Supplementary Material 1. [file 41073_2025_173_MOESM1_ESM.docx]
